# Targeting hepcidin in colorectal cancer triggers a TNF-dependent-gasdermin E-driven immunogenic cell death response

**DOI:** 10.1186/s40164-024-00562-y

**Published:** 2024-09-27

**Authors:** Antonio Di Grazia, Eleonora Franzè, Rachele Frascatani, Federica Laudisi, Teresa Pacifico, Lorenzo Tomassini, Davide Di Fusco, Vincenzo Formica, Giuseppe Sica, Carmine Stolfi, Ivan Monteleone, Giovanni Monteleone

**Affiliations:** 1 Department of Systems Medicine, University of Rome “Tor Vergata”, Via Montpellier, 1, 00133 Rome, Italy; 2 Gastroenterology Unit, Fondazione Policlinico “Tor Vergata”, Rome, Italy; 3 Medical Oncology Unit, Fondazione Policlinico “Tor Vergata”, Rome, Italy; 4 Department of Surgery, University Rome of “Tor Vergata”, Rome, Italy; 5 Department of Biomedicine and Prevention, University of Rome “Tor Vergata”, Rome, Italy

**Keywords:** Anti-cancer immunity, Lytic cell death, Caspases, PD-1

## Abstract

Interactions between colorectal cancer (CRC) cells and the noncancerous cells in the tumor microenvironment (TME) induce mechanisms for the escape of tumor cells from immune attack. Hepcidin, a peptide that controls immune cell functions, is overproduced by CRC cells. This study aimed to evaluate whether hepcidin acts as a regulator of anti-tumor immunity in CRC. Hepcidin silencing in CRC cells was followed by enhanced TNF-driven caspase-dependent cleavage of GSDM E and death. Mice engrafted with hepcidin-deficient CT26 cells developed fewer and smaller tumors than control mice as a result of the action of tumor-infiltrating CD8+ T lymphocytes and were protected from the development of tumors in a vaccination model and exhibited long-lasting tumor protection. Additionally, hepcidin deficiency enhanced the response of mice bearing CT26-derived tumors to anti-PD-1 therapy. These results suggest that targeting hepcidin in CRC cells enhances the production of TNF thereby triggering a caspase/GSDM E-driven lytic cell death with the downstream effect of boosting a robust immune response against tumor antigens.

**Supplementary Information:**

The online version contains supplementary material available at 10.1186/s40164-024-00562-y.

**To the editor:**

Interactions between colorectal cancer (CRC) cells and the noncancerous cells in the tumor microenvironment (TME) induce mechanisms for the escape of tumor cells from immune attack [1]. We and others have recently shown that CRC cells produce high levels of hepcidin, a peptide hormone that acts as an anti-microbial factor and regulator of iron homeostasis through the hepcidin-ferroportin (FPN1) axis [2–4]. Hepcidin induces regulatory molecules in monocytes and suppresses the function of inflammatory macrophages [5, 6], raising the possibility that CRC cell-derived hepcidin can contribute to generating a TME that promotes the escape of neoplastic cells from immune attack. This study aimed to assess whether hepcidin is a negative regulator of anti-cancer immunity in CRC.

Hepcidin silencing triggered HCT116 cell death and this was prevented by exogenous hepcidin (Fig. S1A, Fig. 1A). Hepcidin-deficient cells showed enhanced SYTOX green uptake, secretion of HMGB1 (Fig. 1B, C), and cleavage of gasdermin (GSDM) E (Fig. 1D, Fig. S1B). GSDM E silencing in HCT116 cells reduced the rate of hepcidin-deficient cell death (Fig. 1E; Fig. S1C, D). GSDM E is silenced in various tumor cell types due to high methylation of the GSDM E promoter region [7]. Hepcidin silencing did not kill the GSDM E-deficient SW480 and AGS cells unless these cells were treated with decitabine, a cytosine analog that acts as a DNA methyltransferase inhibitor to enhance GSDM E expression (Fig. S1E-H).

**Fig. 1:**
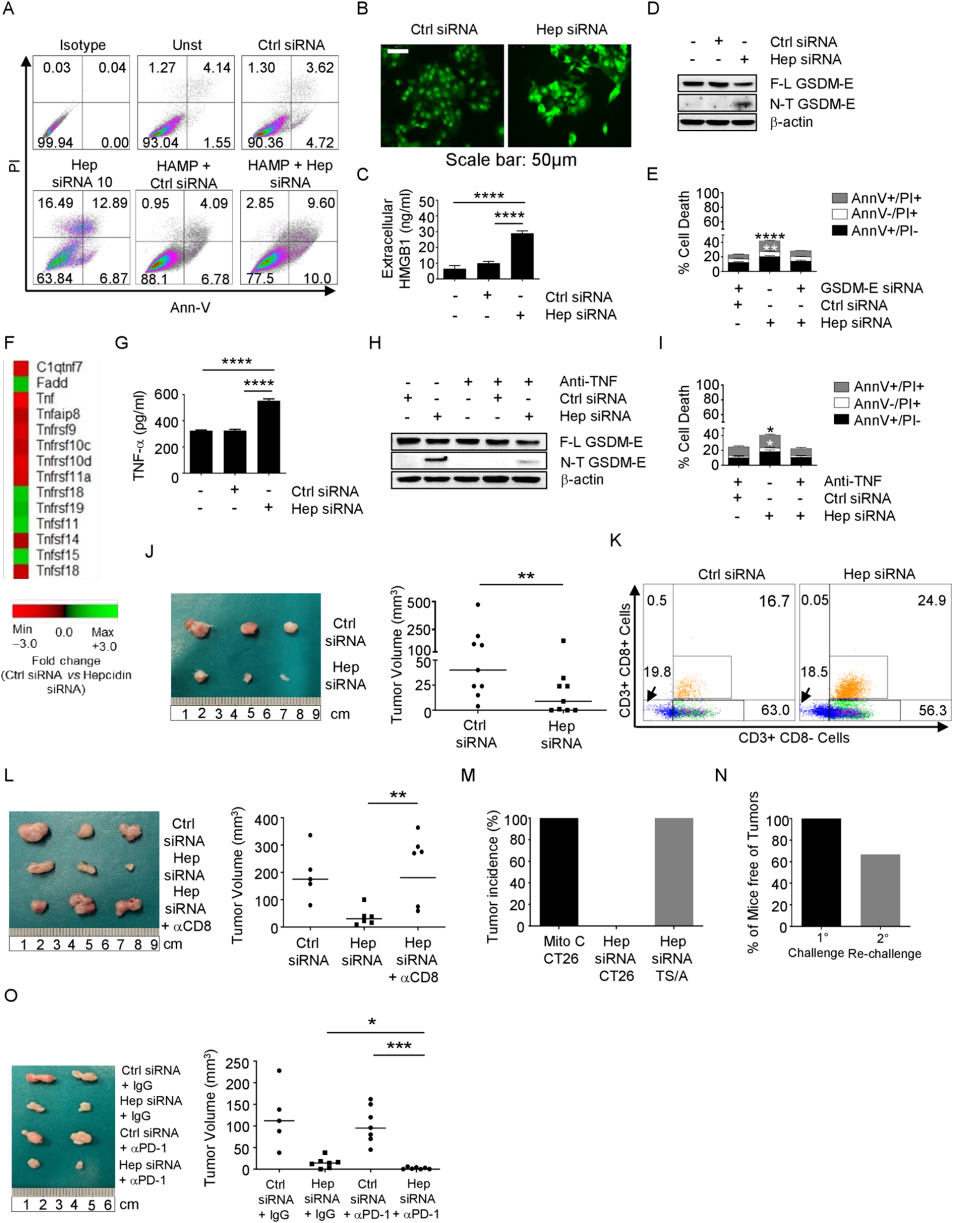
**A** Representative dot-plots showing the percentages of AV- and/or PI-positive HCT116 cells either left untreated or transfected with a control or hepcidin siRNA for 48 h in the presence or absence of exogenous hepcidin (HAMP) (1000 ng/mL). One of 5 separate experiments in which similar results were obtained is shown. **B**, **C** SYTOX green uptake and levels of HMGB1 in the culture supernatants of HCT116 cells either left untreated or transfected with a control or hepcidin siRNA (25 nmol/L) for 48 h. Data are expressed as mean ± SD of three experiments. ****p < 0,0001. **D** Representative Western blots showing the full length (F-L) and cleaved (N-T) GSDM E and β-actin in HCT116 cells either left untreated or transfected with a control or hepcidin siRNA (25 nmol/L) for 48 h. **E** Histograms showing the percentages of AV- and/or PI-positive HCT116 cells transfected with either a hepcidin siRNA (25 nmol/L) or co-transfected with control siRNA/hepcidin siRNA (25 nmol/L) plus GSDM-E siRNA (5 nmol/L) for 48 h. Data are expressed as mean ± SD of all experiments. Hepcidin siRNA vs hepcidin siRNA plus GASDM-E siRNA, **p < 0.01; ****p < 0,0001. **F** Heat map showing microarray-based differential expression, log2 (fold change) of TNF-related genes in HCT116 transfected with a control or hepcidin siRNA (25 nmol/L) for 48 h. **G** Histograms showing the levels of TNF protein in HCT116 either left untreated or transfected with a control or hepcidin siRNA (25 nmol/L) for 48 h; ****p < 0,0001. **H** Representative Western blots showing F-L GSDM E, N-T GSDM E, and β-actin. One of 4 separate experiments in which similar results were obtained is shown. **I** Histograms showing the percentages of AV- and/or PI- positive HCT116; data are expressed as mean ± SD of all experiments. *p < 0,05. **J** Representative images and relative graphs showing the volume of CT26-derived tumors in BALB/c mice. CT26 cells were transfected with either a control siRNA or hepcidin siRNA (25 nmol/L) for 36 h and subcutaneously injected into the left flank of mice (1 × 10^6^ per mouse) (day 0). Tumor growth was monitored until sacrifice (day 13). Each point in the graph represents the value of the tumor volume in each mouse. **p < 0,01. **K** Representative dot-plots showing the percentages of CD3+ CD8+ and CD3+ CD8− cells from CT26-derived tumors. One of 2 experiments in which 8 mice per group were analyzed is shown. **L** Representative images and the relative graph showing the volume of CT26-derived tumors. CT26 were transfected with either a control siRNA or hepcidin siRNA (25 nmol/L) for 36 h and subcutaneously injected into the left flank of BALB/c mice (1 × 10^6^ per mouse) (day 0). CD8+ cell depletion was made with intraperitoneal injection of α-CD8 (100 µg per mouse). Tumor growth was monitored until sacrifice (day 13). Each point in the graph represents the value of tumor volume in each mouse. **p < 0,01. **M** Tumor incidence following injection of CT26 or TS/A cells in mice that were previously vaccinated with hepcidin siRNA-transfected CT26 cells. Mitomycin-treated cells were injured at the same of vaccination as a positive control. **N** The percentage of mice free of tumors at day 26 after the vaccination protocol as described in L and injected again into the right flank with CT26. **O** Representative images and the relative graph showing the volume of CT26-derived tumors in BALB/c mice injected with CT26 cells transfected with either a control or hepcidin siRNA (25 nmol/L) for 36 h and then subcutaneously injected into the left flank. PD-1 blockade was made with intraperitoneal injection of α-PD-1 (100 µg per mouse). Each point in the graph represents the value of tumor volume in each mouse; *p < 0,05; ***p < 0.001

GSDM-mediated cell death is triggered by caspases [8, 9]. Hepcidin silencing increased caspase 8 and caspase 3 activation (Fig. S1I, J). Qvad, a pan-caspase inhibitor, blocked hepcidin silencing-driven GSDM E cleavage (Fig. S1K).

A gene array showed that hepcidin silencing changed TNF-α signaling-related gene expression. Specifically, hepcidin-silenced cells had enhanced TNF-α levels (Fig. 1F), a finding that was confirmed by ELISA of extracts of HCT116 cells and primary CRC tissues (Fig. 1G; Fig. S2A). Neutralization of endogenous TNF-α in hepcidin-silenced HCT116 cells reduced caspase 8 activation (Fig. S2B), GSDM E cleavage (Fig. 1H), and the rate of cell death (Fig. 1I). Consistently, TNF-α enhanced caspase 8 activation (Fig. S2C), GSDM E cleavage (Fig. S2D), and induction of death in HCT116 cells (Fig. S2E).

Since hepcidin activates Stat3 [2] and Stat3 represses TNF-α transcription in immune cells [10], we evaluated whether TNF-α induction in hepcidin-deficient CRC cells relies on Stat3 inactivation. Hepcidin silencing in HCT116 cells reduced Stat3 phosphorylation (Fig. S2F). The gene array showed that hepcidin-silenced HCT116 cells had reduced levels of several Stat3 signaling-related genes (Fig. S2G). Knockdown of Stat3 in HCT116 cells prevented the hepcidin-mediated inhibition of TNF-α expression (Fig. S2H, I), confirming the involvement of STAT3 in the hepcidin-induced TNF-α reduction. Stimulation of STAT3-deficient cells with hepcidin increased TNF-α expression, suggesting that, in the absence of STAT3, hepcidin can activate further pathways regulating TNF-α expression.

In line with human data, hepcidin silencing in mouse CT26 cells enhanced cleavage of GSDM E and death (Fig. S3A, B). Then, we examined whether cancer cell-derived hepcidin controls the in vivo anti-cancer immune response. Tumors induced by grafting BALB/c mice with CT26 cells transfected with hepcidin siRNA grew significantly less than tumors expressing hepcidin (Fig. 1J). The microenvironment of hepcidin-silenced CT26-derived tumors had higher percentages of CD8+ T cells and of granzyme B- or perforin-positive CD8+ T lymphocytes, while the fractions of both CD3+ CD8− T cells and NK cells were unchanged (Fig. 1K and Fig. S3C, D). Depletion of CD8+ T cells (Fig. S3E) abrogated the inhibitory effect of hepcidin silencing on the volume of CT26-derived tumors (Fig. 1L).

Next, we assessed whether hepcidin deficiency promotes an activated immunogenic cell death. For this purpose, mice were injected subcutaneously in the left flank with control or hepcidin-silenced CT26 cells and challenged on the right flank 1 week later with wild-type CT26 cells. Following re-challenging, tumors developed in 11/11 control mice and none of those vaccinated with hepcidin-silenced cells (Fig. S3F). In contrast, the vaccination with hepcidin silenced-CT26 cells did not prevent the development of tumors induced by TS/A cells, a murine mammary adenocarcinoma cell line, which expresses a repertoire of antigens different from those of CT26 cells (Fig. 1M and Fig. S3G) [11]. Most animals not developing tumors after the challenge with hepcidin-silenced CT26 cells were protected against a second inoculation of CT26 cells (Fig. 1N).

CRC exhibits a low response rate to immunotherapy due to the immunosuppressive TEM [12]. Since lytic cell death enhances anticancer immunity [13], we assessed whether hepcidin-deficient CRC cells responded adequately to immunotherapy. CT26 cells, which are microsatellite stable (MSS) and unresponsive to PD-1 neuralization [14], were transfected with control or hepcidin siRNA and subcutaneously implanted into mice that were then treated with a blocking PD1 antibody (αPD-1) or control IgG. There was a significant reduction of the tumor volume and increased fraction of TNF-expressing CD3+ CD8+ cells in mice engrafted with hepcidin-deficient CT26 cells and treated with αPD-1 as compared to the other groups (Figs. 1O, 2A, B).

**Fig. 2:**
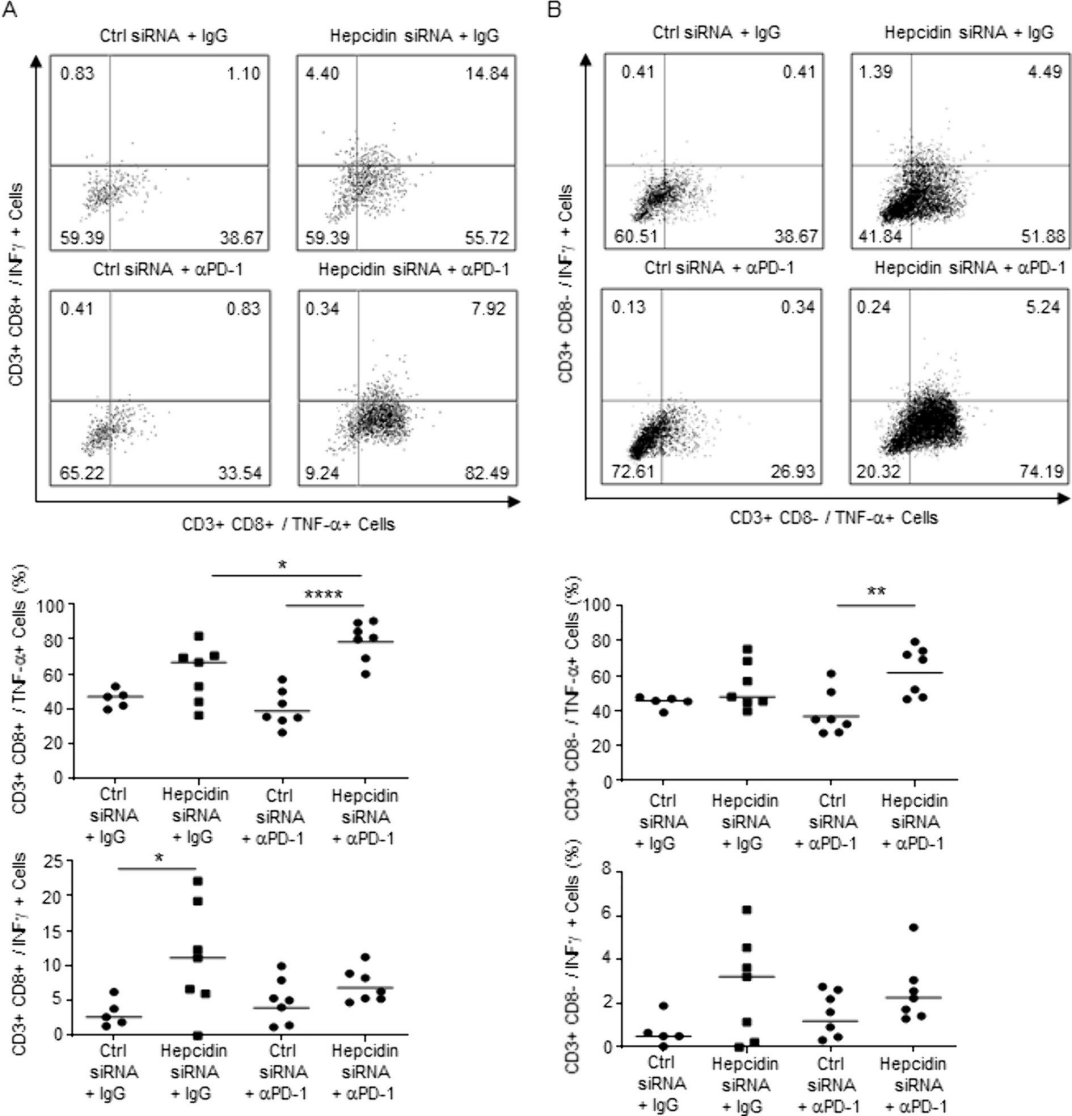
**A**, **B** Representative dot-plots and the corresponding graphs showing the percentages of CD3+ CD8+ and CD3+ CD8− cells producing cytokines in CT26-derived tumors taken from BALB/c mice intraperitoneally injected with α-PD-1 or IgG (both 100 µg per mouse). Each point in the graph represents the value of each mouse. *p < 0,05; **p < 0.01; ****p < 0.0001

Our results delineate a mechanism by which high hepcidin sustains CRC and suggest the use of hepcidin inhibitors in the treatment of cancer patients. Although such drugs appear to be well-tolerated and safe [15], further studies are needed to validate their use in clinical settings.

## Supplementary Information

Supplementary Material 1

Supplementary Material 2

Supplementary Material 3

Supplementary Material 4

## Data Availability

Data is provided within the manuscript and supplemental information files.

## Abbreviations

HMGB1: High mobility group protein 1
ICD: Immunogenic cell death
CRC: Colorectal cancer
FPN1: Ferroportin
AV: Annexin-V
PI: Propidium iodide
GSDM: Gasdermin
ICIs: Immune checkpoint inhibitors
TME: Tumor microenvironment
MSS: Microsatellite stable
MCI: Microsatellite instability
PD-1: Programmed cell death protein 1 antibody-1
