# Supplementary figures and images for: Targeting hepcidin in colorectal cancer triggers a TNF-dependent-gasdermin E-driven immunogenic cell death response
Source: Exp Hematol Oncol. 2024 Sep 27;13:95. doi: 10.1186/s40164-024-00562-y (PMC11437719; doi:10.1186/s40164-024-00562-y)

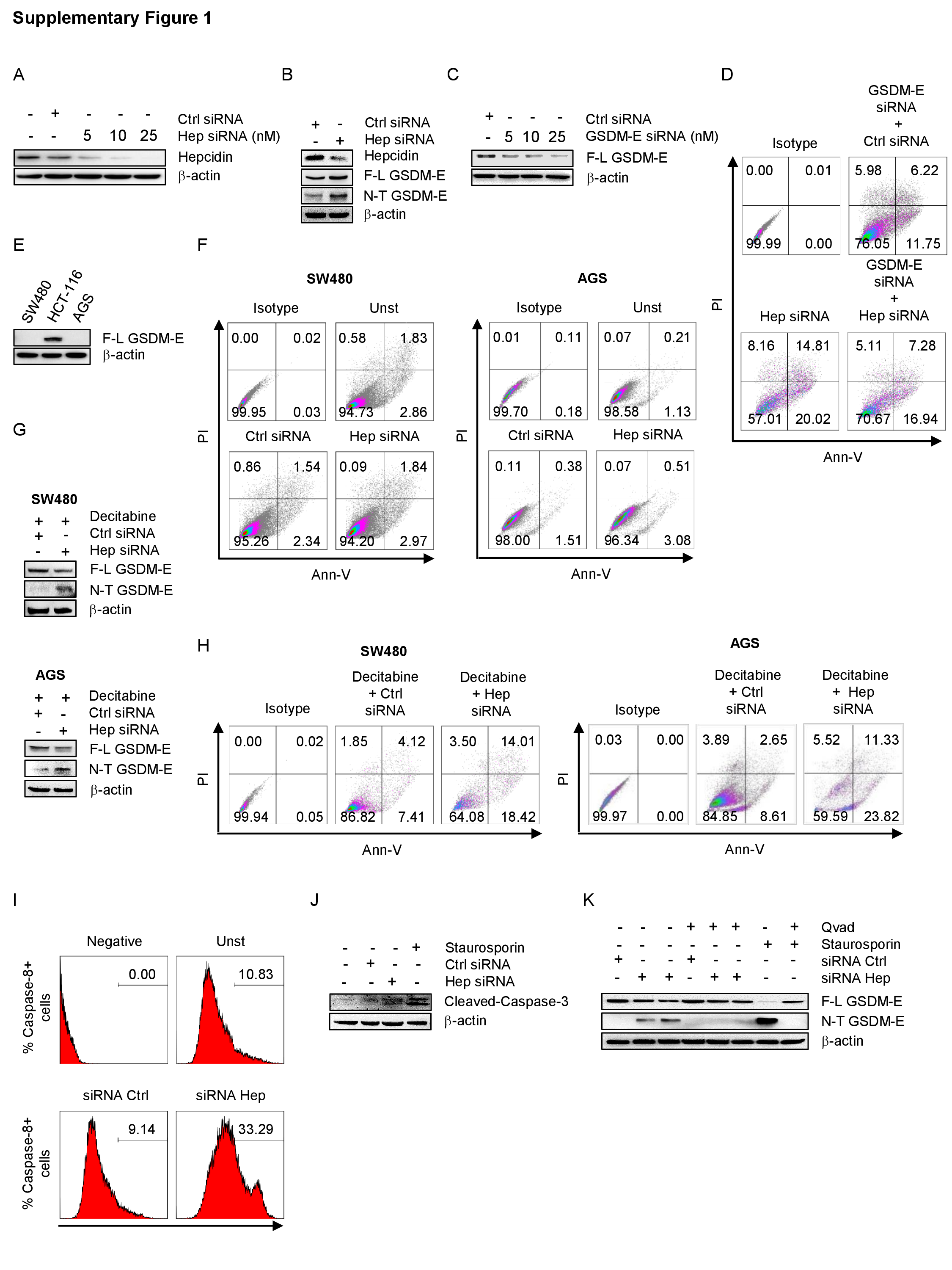

Supplement: Supplementary file 1 — Supplementary Material 1 [file 40164_2024_562_MOESM1_ESM.tiff]

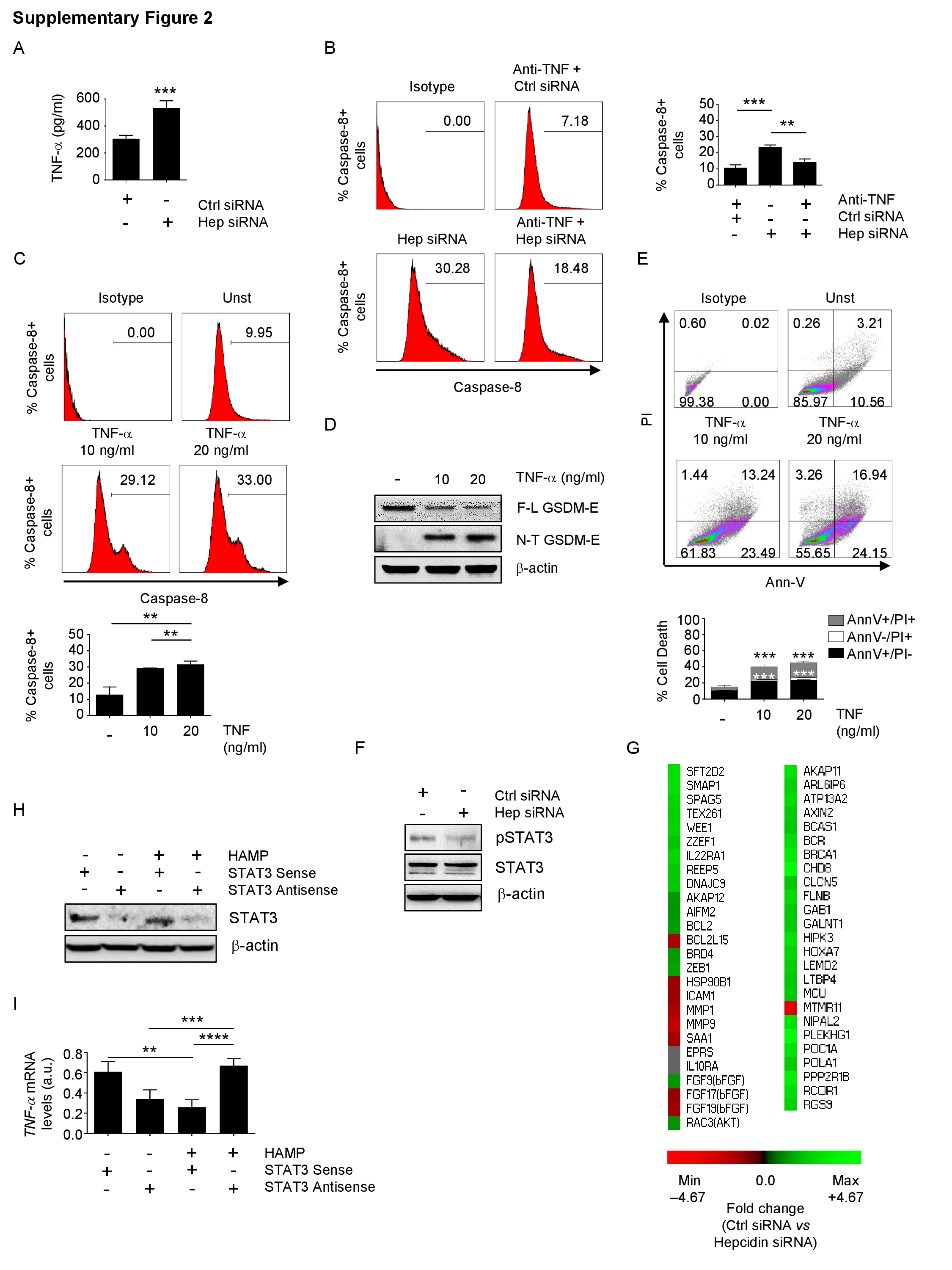

Supplement: Supplementary file 2 — Supplementary Material 2 [file 40164_2024_562_MOESM2_ESM.tiff]

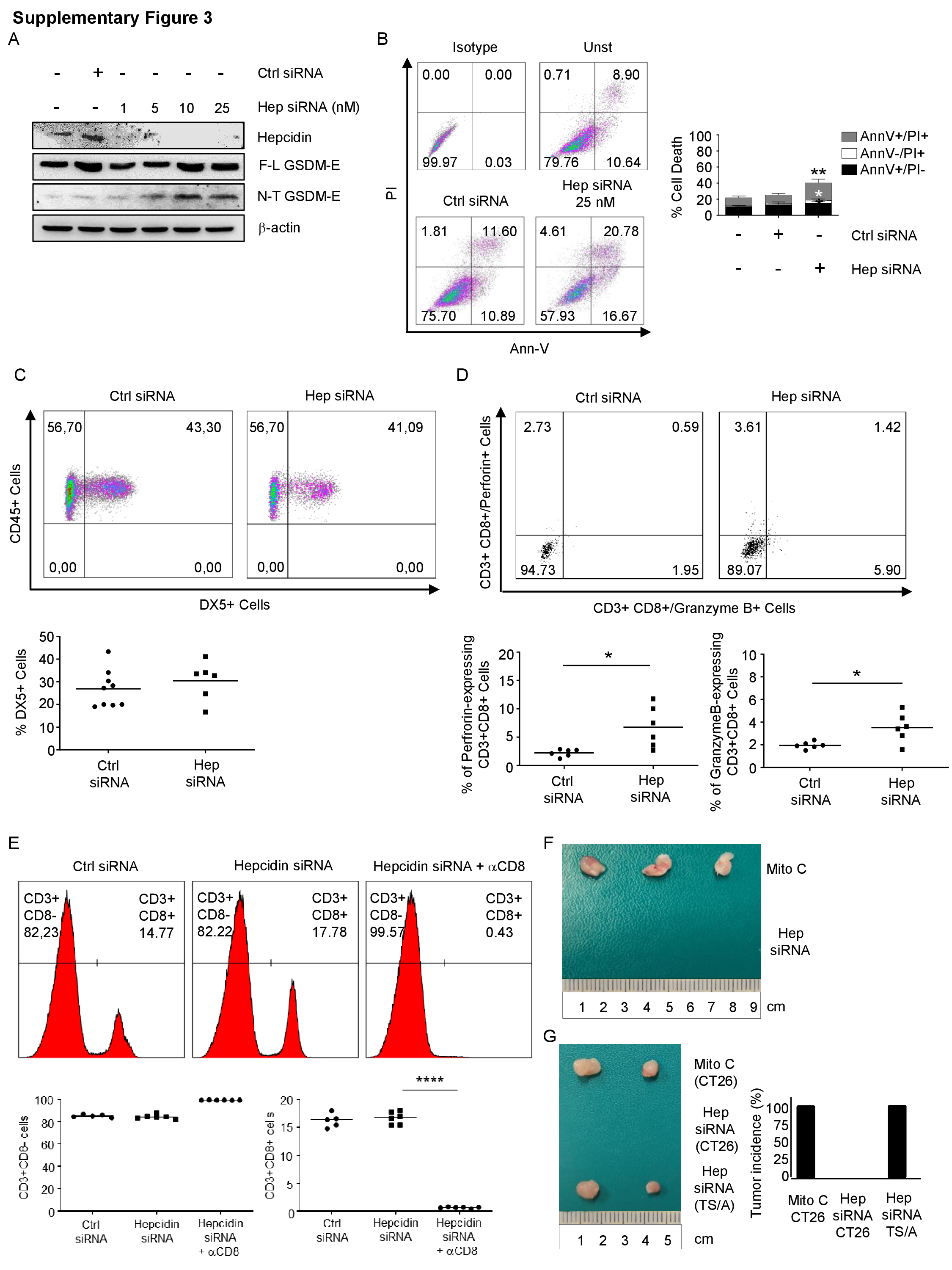

Supplement: Supplementary file 3 — Supplementary Material 3 [file 40164_2024_562_MOESM3_ESM.tiff]
