# Supplementary material for: Targeting hepcidin in colorectal cancer triggers a TNF-dependent-gasdermin E-driven immunogenic cell death response
Source: Exp Hematol Oncol. 2024 Sep 27;13:95. doi: 10.1186/s40164-024-00562-y (PMC11437719; doi:10.1186/s40164-024-00562-y)
Supplement: Supplementary file 4 — Supplementary Material 4 [file 40164_2024_562_MOESM4_ESM.pdf]

SUPPLEMENTARY INFORMATION

TARGETING HEPCIDIN IN COLORECTAL CANCER TRIGGERS A TNF-DEPENDENT-GASDERMIN E-DRIVEN IMMUNOGENIC CELL DEATH RESPONSE

Antonio Di Grazia[1]; Eleonora Franzè[1]; Rachele Frascatani [1], Federica Laudisi[1]; Teresa Pacifico[1]; Lorenzo Tomassini[1]; Davide Di Fusco[1]; Vincenzo Formica[3]; Giuseppe Sica[4]; Carmine Stolfi[1]; Ivan Monteleone[5] and Giovanni Monteleone[1,2,*].

[1] Department of Systems Medicine, University of Rome "Tor Vergata", 00133 Rome, Italy; [2] Gastroenterology Unit, Fondazione Policlinico "Tor Vergata", Rome, Italy; [3] Medical Oncology Unit, Fondazione Policlinico "Tor Vergata", Rome, Italy; [4] Department of Surgery, University Rome of "Tor Vergata", Rome, Italy; [5] Department of Biomedicine and Prevention, University of Rome "Tor Vergata", Rome, Italy.

Running title: Hepcidin and colorectal cancer

Acknowledgments: Funding: This work was supported by PRIN HEAL ITALIA - PE6 (PE_00000019) and PNRR-MCNT2-2023-12377866.

The authors wish to thank Nogra Pharma and PPM Services.

Address for correspondence:

Giovanni Monteleone

Department of Systems Medicine

University of Rome "TOR VERGATA"

Via Montpellier, 1

00133 Rome, Italy

Phone: +39.06.72596158

Fax: +39.06.72596391

Email: Gi.Monteleone@Med.uniroma2.it

Keywords: anti-cancer immunity, pyroptosis, caspases, PD-1.

Methods

Cell Culture

All reagents were from Sigma-Aldrich (Milan, Italy) unless specified. HCT116, SW480, CT26, and AGS were purchased from the American Type Culture Collection (ATCC, Manassas, VA, USA) while TS/A was obtained from (Merck, Milan, Italy). Cells were maintained in McCoy's 5A (HCT116), RPMI 1640 (SW480 and CT26), or Dulbecco's modified Eagle's high glucose medium (AGS, and TS/A) supplemented with 10% fetal bovine serum (FBS, Lonza, Verviers, Belgium) 1% P/S and 4 mM L-glutamine in a 37 ◦C, 5% CO2, fully humidified incubator. Cell lines were authenticated by STR DNA fingerprinting using the PowerPlex 18D System kit, according to the manufacturer's instructions (Promega, Milan, Italy). The STR profiles of all the cell lines matched the known DNA fingerprints. For silencing studies, 2.5×10^5 cells were transfected with human or murine hepcidin siRNA (1-25 nmol/L;) or control siRNA (final concentration 25 nmol/L; both from Santa Cruz Biotechnology, Santa Cruz, CA, USA) using Opti-MEM medium and Lipofectamine 3000 reagent (both from Life Technologies, Milan, Italy) according to the manufacturer's instructions. Cell morphology changes were evaluated by light microscopy (Nikon Eclipse TE200-S, Tokyo, Japan) and photographed. For sytox green uptake assay, HCT-116 cells transfected with control or hepcidin siRNA were incubated with sytox (final concentration 1 µmol/L; Thermo Fisher Scientific, Monza, Italy) 6h before the and of transfection The images were visualized using a Leica DMI4000 B microscope with Leica application suite software (V4.6.2) (Wetzlar, Germany).

In parallel experiments, cells transfected with hepcidin siRNA were cultured with exogenous hepcidin (HAMP) (final concentration 1000 ng/mL; Creative Biomart, Shirley, NY,

USA), while AGS and SW480 cells were treated with decitabine (5 μmol/L) for 48 h and then either analyzed for GSDM E expression or transfected for further 48h with control siRNA or hepcidin siRNA. HCT116 cells (2.5 × 10^5) were also transfected with GSDM E siRNA (5-25 nmol/L; Santa Cruz Biotechnology). To investigate whether GSDM E is involved in the hepcidin silencing-induced cell death, HCT-116 (2.5 × 10^5) were co-transfected with GSDM E siRNA (5 nmol/L) plus hepcidin siRNA (25 nmol/L) for 48 h. To assess the involvement of TNF-α in the hepcidin silencing-driven cell death, a neutralizing anti-TNF or control IgG (final concentration 2.5 nmol/L; Thermo Fisher Scientific) was added to cultures of HCT116 cells transfected with hepcidin siRNA. Additionally, HCT116 cells (2.5 × 10^5) were stimulated with recombinant human TNF (10-20 ng/mL; R&D Systems) for 24 h. To ascertain whether hepcidin controls TNF expression through the Stat3 pathway, HCT-116 (2.5 × 10^5) cells were transfected with either Stat3 sense or Stat3 antisense oligonucleotides (both used at 100 nM) in the presence or absence of exogenous hepcidin (1000 ng/mL) for 24h. The phosphorothioate single-stranded oligonucleotide (5'-AGCTGATTCCATTGGGCCAT-3') matching the human Stat3 complementary DNA sequence was synthesized in both the sense and antisense orientations (Integrated DNA Technologies Leuven, Belgium). As a positive control of the activation of caspase 3, we used HCT116 cells stimulated with staurosporin (1 μmol/L) for 24h.

Organ culture

Fresh human CRC explants were obtained from patients who had undergone colonic resection for sporadic CRC at the Tor Vergata University Hospital (Rome, Italy). Each patient who took part in the study gave written informed consent and the study protocol was approved by the local Ethics Committees (Tor Vergata University Hospital, Rome (129/17). CRC explants were placed on Millicell inserts (EMD Millipore) in a 6-well plate containing RPMI-1640 medium

supplemented with 10% FBS, 1% P/S, and 50 μg/mL gentamycin. Organ culture transfection with either hepcidin siRNA or control siRNA (both used at 100 nM) for 24 h was performed using Opti-MEM medium and Lipofectamine 3000 reagent according to the manufacturer's instructions. The culture was performed in an organ culture chamber at 37 °C in a 5% CO_2/95% O_2 atmosphere.

Protein Extraction and Western Blotting

Total proteins were isolated in lysis buffer containing 0.1 mmol/L EDTA, 10 mmol/L HEPES (pH 7.9), 10 mmol/L KCl, 0.5% Nonidet P40, 0.2 mmol/L ethylene glycol-bis (β-aminoethyl ether)-N,N,N', N'-tetraacetic acid and complemented with 10 mg/mL leupeptin, 1 mmol/L dithiothreitol, 10 mg/mL aprotinin, 1 mmol/L phenylmethylsulfonyl fluoride, 1 mmol/L NaF and 1 mmol/L Na3VO4. Lysates were clarified by centrifugation and analyzed by sodium dodecyl sulfate-polyacrylamide gel electrophoresis (SDS-PAGE). Membranes were then incubated with antibodies against human or mouse hepcidin, and human GSDM E, (all final dilution 1:1000, Abcam, Cambridge, UK), caspase-3, pStat3 Tyr705 (final dilution 1:1000, Cell Signaling Technology, Danvers, MA), and human total Stat3 (final dilution 1:1000, Santa Cruz Biotechnology) followed by a secondary antibody conjugated to HRP (Dako, Agilent Technologies, Santa Clara, CA, USA). A mouse anti-β-actin antibody (final dilution 1:1000, Santa Cruz Biotechnology) was used to detect β-actin.

Assessment of HMGB1

HMGB1 was quantitated in the culture supernatants of HCT116 cells transfected with control or hepcidin siRNA for 48 h using an enzyme-linked immunosorbent assay (IBL International GmbH, Hamburg, Germany) according to the manufacturer's instructions.

RNA extraction, RT-PCR, and Transcriptome Analysis

RNA was extracted from HCT116 cells and CRC explants transfected with either control or hepcidin siRNA using the PureLink mRNA mini kit (Thermo Fisher Scientific, Waltham, MA, USA), according to the manufacturer's instructions. RNA (1 μg per sample) was reverse transcribed into complementary DNA (cDNA) by M-MLV Reverse Transcriptase and this was amplified using the following conditions: denaturation for 1′ minute at 95 °C; annealing for 30 s at 62°C for human TNF at 60 °C for β-actin; extension at 72 °C for 30 s. RNA expression was calculated relative to the housekeeping β-actin gene on the base of the ΔΔCt algorithm. Sequences of the primers were as follows: TNF-α: forward, 5′-AGGCGGTGCTTGTTCCTCAG-3′ and reverse, 5′- GGCTACAGGCTTGTCACTCG-3′; β-actin: Forward: 5′-AAGATGACCCAGATCATGTTTGAGACC-3′; Reverse: 5′-AGCCAGTCCAGACGCAGGAT-3′.

HCT116 samples with quantified complementary DNA (cDNA) were sequenced in the Microarray Unit of the Consortium for Genomic Technologies (Milan, Italy) by hybridization to GeneChip Q21 Human Gene 2.0 ST microarrays. Transcripts were selected based on a fold change value of 2 or higher. All the transcripts present on the GeneChip array were mapped to related classes by Gene Ontology, which provided the fold change generated from the comparison Q23 between control siRNA vs hepcidin siRNA.

TNF-α enzyme-linked immunosorbent assay

Human TNF-α was assessed in total proteins extracted from HCT116 cells or CRC explants transfected with either control or hepcidin siRNA by a commercial ELISA kit (R&D Systems, Minneapolis, MN, USA) according to the manufacturer's instructions.

Mouse tumors models

Female BALB/c mice 6 weeks old were obtained from the Charles River Laboratories (Lodi, Italy) and maintained in filter-topped cages on autoclaved food and water at the University of Rome "Tor Vergata" animal facility (Rome, Italy). All animal experiments were approved by the local Institutional Animal Care and Use Committee (authorization 777/2023-PR), registered with the Italian Ministry of Health). CT26 cells transfected with either control or hepcidin siRNA (25 nmol/L) for 36 h were subcutaneously implanted into the left flank of immunocompetent BALB/c mice (1×10^6 per mouse) whose fur was previously shaved and depilated (day 0). Tumor growth was monitored until sacrifice (day 13). Tumor volume was calculated using the formula: long diameter × short diameter x depth diameter/2. Tumor-infiltrating cells were isolated from the tumor samples by enzymatic digestion with Liberase TM (200 µg/mL) and DNase I (200 µg/mL) (both Roche Diagnostics GmbH, Mannheim, Germany) and analyzed by flow cytometry. Depletion of CD8+ cells was achieved by injecting mice intraperitoneally with anti-mouse CD8 antibodies (α-CD8) (100 µg per mouse; Biolegend, France) 4 times starting 2 days before CT26 injection and then every 4 days. In the end, after red blood cell lysis with a commercial buffer (Roche Diagnostic GmbH), splenic cells were analyzed by flow cytometry.

To evaluate whether the anti-cancer immune response induced by hepcidin-silenced CT26 cells was amplified by PD1 blockade, mice implanted with either control or hepcidin siRNA-transfected CT26 were intraperitoneally injected with an anti-PD-1 antibody (αPD-1) or control IgG (100 µg per mouse) (both from Bio x cell, Lebanon, NH, USA) four times starting 3 days after CT26 injection and then every 3 days. To study the potential and specificity of the hepcidin silencing-induced immune response, CT26 cells were transfected with hepcidin siRNA (25 nmol/L) or treated with Mitomycin C (100 µg/mL), and after 36 h were subcutaneously implanted

into the left flank of immunocompetent BALB/c mice (1×10^6 per mouse). One week later, the mice were injected with living CT26 or living TS/A (1×10^6 per mouse) into the contralateral flank. Mice were monitored for tumor growth until sacrifice (day 13). Tumor volume was calculated as described above. In parallel experiments, mice that did not develop tumors at day 13 following the vaccination with hepcidin-silenced CT26 were re-challenged with living CT26 (1×10^6 per mouse) and then monitored for tumor growth for a further 13 days (day 26).

Flow cytometry

Cells were collected, washed 2 times in AV buffer, stained with FITC-AnnV (final dilution 1:100; Immunotools, Friesoyte, Germany) according to the manufacturer's instructions, and incubated with 5 mg/mL PI for 30 minutes at 4°C. The percentage of active caspase-8-expressing cells was quantified using a specific antibody (FITC-IETD-FMK ; final dilution 1:100; Biovision, Milpitas, CA, USA). To characterize the fractions of tumor-infiltrating cells in CT26-derived tumors, cells were stimulated with phorbol myristate acetate (10 ng/mL), ionomycin (1 μg/mL), and brefeldin A (10 μg/mL; eBioscience) for 4 h. To discriminate live from dead cell populations, a live/dead cell assay was performed (final dilution 1:1000, Life technologies). Cells were then washed and stained with the following monoclonal antibodies: CD45-APC-H7, CD3-Pacific blue, CD8 FITC, DX5 Pe, IFN-γ FITC, TNF-α Pe (all at 1:50 final dilution, Becton Dickinson, Milan Italy), Granzyme B Pe-Cy7 (1:50 final dilution, Biolegend, San Diego, CA, USA), Perforin APC (1:50 final dilution, Invitrogen, Waltham, MA, USA), or respective isotype control antibodies. The fluorescence signals were assessed using the Kaluza software (Beckman Coulter Life Sciences, Pasadena, CA, USA).

Statistical Analysis

Statistical analysis of the data was performed by using the Student t-test or Tukey's multiple comparisons test. GraphPad Prism 6 (GraphPad Software, La Jolla, CA, USA) was used for statistical and graphical data evaluations. P values < 0.05 were considered statistically significant.

List of abbreviations: immunogenic cell death (ICD); colorectal cancer (CRC); ferroportin (FPN1); annexin-V (AV); propidium iodide (PI); high mobility group protein 1 (HMGB1); gasdermin (GSDM); immune checkpoint inhibitors (ICIs); tumor microenvironment (TME); microsatellite stable (MSS); microsatellite instability (MCI)

Figure legends

Suppl. Figure 1. A. Representative Western blots showing hepcidin and β-actin in HCT116 cells either left untreated or transfected with a control (25 nmol/L) or hepcidin siRNA (5; 10 and 25 nmol/L) for 48 h. One of 6 experiments in which similar results were obtained is shown. **B.** Representative Western blots showing hepcidin, F-L GSDM E, N-T GSDM E, and β-actin in human primary CRC explants transfected with either a control or hepcidin siRNA (100 nmol/L) for 24 h. **C**. Representative Western blots showing F-L GSDM E and β-actin in HCT116 cells either left untreated or transfected with a control or hepcidin siRNA (25 nmol/L) for 48 h. **D**. Representative dot-plots showing the percentage of AV- and/or PI-positive HCT116 cells either transfected with hepcidin siRNA (25 nmol/L) or co-transfected with control siRNA or hepcidin siRNA (25 nmol/L) (25 nmol/L) plus GSDM E siRNA (5 nmol/L) (5 nmol/L) for 48 h. **E**. Representative Western blots showing F-L GSDM E and β-actin in SW480, HCT116 and AGS cells. **F**. Representative dot-plots of AV- and/or PI-positive SW480 and AGS cells either left untreated or transfected with a control or hepcidin siRNA (25 nmol/L) for 48 h. **G**. Representative Western blots showing F-L GSDM E and β-actin in SW480 and AGS stimulated with decitabine (5 μmol/L) for 72 h. **H**. Representative dot-plots showing AV- and/or PI-positive SW480 and AGS transfected with a control or hepcidin siRNA (25 nmol/L) in the presence of decitabine for 48 h. **I**. Representative dot-plots of active caspase-8-expressing HCT116 cells either left untreated or transfected with either a control or hepcidin siRNA (25 nmol/L) for 48 h. **J**. Representative Western blots showing cleaved-caspase-3 and β-actin in HCT116 cell line left untreated or transfected with either a control or hepcidin siRNA (25 nmol/L) for 48 h. Staurosporin was used

as a positive control. **K**. Representative Western blots showing F-L GSDM E, N-T GSDM E and β-actin in HCT116 cells transfected with either a control or hepcidin siRNA (25 nmol/L) in the presence or absence of Q-vad for 48 h.

Suppl. Figure 2. A. Histograms showing the levels of TNF primary CRC explants transfected with either a control or hepcidin siRNA (100 nmol/L) for 24 h; ***p <0,001. **B**. Representative dot-plots and the corresponding histograms showing the percentages of active caspase-8 of HCT116 either transfected with a control or hepcidin siRNA (25 nmol/L) in the presence or absence of anti-TNF (2,5 nmol/L) for 48 h. Data are expressed as mean ± SD of all experiments. **p< 0,01; ***p< 0,001. **C-E**. HCT-116 were left untreated or stimulated with TNF (10 or 20 ng/mL) for 48 h. Representative dot-plots and the corresponding histograms showing the percentages of cells expressing active caspase-8 (C). Representative Western blots showing F-L GSDM E; N-T GSDM E and β-actin (D). Representative dot-plots and the corresponding histograms showing the percentages of AV- and/or PI-positive HCT116 either left untreated or stimulated with TNF-α (10 or 20 ng/mL) for 48 h. Data are expressed as mean ± SD of all experiments. ***p< 0,001 (E). **F**. Representative Western blots showing pSTAT3, STAT3, and β-actin in HCT116 transfected with either a control siRNA or hepcidin siRNA (25 nmol/L) for 24 h. **G**. Heat map showing microarray-based differential expression, log2 (fold change) of STAT3-regulated genes in HCT116 transfected with either a control siRNA or hepcidin siRNA (25 nmol/L) for 48 h. **H**. Representative Western blots showing STAT3 and β-actin in HCT116 transfected with either a STAT3 Sense or STAT3 Antisense oligonucleotide (100 nmol/L) in the presence or absence of exogenous hepcidin (HAMP) (1000 ng/mL) for 24 h. **I**. Representative histograms showing TNF transcripts in HCT116 transfected with either a STAT3 sense or STAT3 antisense (100 nmol/L) in the presence or absence of exogenous hepcidin (HAMP) (1000 ng/mL)

for 24 h. TNF mRNA was normalized to β-actin. Values are expressed in arbitrary units (a.u.) and indicate the mean ± SD of all experiments. **p<0,01; ***p<0,001; ****P<0,0001.

Suppl. Figure 3. A. Representative Western blots showing hepcidin, F-L-GSDM E, N-T-GSDM E and β-actin in CT26 cells left untreated or transfected with either a control siRNA (25 nmol/L) or hepcidin siRNA (1; 5; 10 and 25 nmol/L) for 48 h. **B**. Representative dot-plots and the corresponding histograms showing the percentage of cell death assessed by flow-cytometry analysis of AV- and/or PI-positive CT26 cells left untreated or transfected with either control or hepcidin siRNA (25 nmol/L) for 48 h. Data are expressed as mean ± SD of all experiments. Untreated or control siRNA transfected cells vs hepcidin siRNA 25 nmol/L transfected cells, *p< 0,05; **p<0,01. **C**. Representative dot-plots and the corresponding graph showing the percentages of DX5+ cells from CT26-derived tumors taken from BALB/c mice. Each point in the graph represents the value for each mouse. **D**. Representative dot-plots and the corresponding graphs showing the percentages of Perforin-expressing CD3+CD8+ and granzyme B-expressing CD3+CD8+ cells from CT26-derived tumors. Each point in the graphs indicates the value for each mouse. *p<0,05. **E**. Representative dot-plots showing the percentage of CD3+ CD8- cells or CD3+ CD8+ cells of splenocytes from BALB/c mice with CT26-derived tumors. The lower panel shows the results relative to all experiments. **F**. Representative images of tumors developing in mice injected with CT26 cells following a vaccination protocol with mitomycin (mito) C-treated CT26 or hepcidin siRNA-transfected cells. **G**. Representative images of tumors following injection of CT26 or TS/A cells in mice that were previously vaccinated with hepcidin siRNA-transfected CT26 cells. The right panel shows the tumor incidence in each group.
